# Theaflavin 3-gallate inhibits the main protease (M^pro^) of SARS-CoV-2 and reduces its count in vitro

**DOI:** 10.1038/s41598-022-17558-5

**Published:** 2022-07-30

**Authors:** Mahima Chauhan, Vijay Kumar Bhardwaj, Asheesh Kumar, Vinod Kumar, Pawan Kumar, M. Ghalib Enayathullah, Jessie Thomas, Joel George, Bokara Kiran Kumar, Rituraj Purohit, Arun Kumar, Sanjay Kumar

**Affiliations:** 1 Biotechnology Division, CSIR-Institute of Himalayan Bioresource Technology, Palampur, Himachal Pradesh 176061 India; 2 Academy of Scientific and Innovative Research, Ghaziabad, Uttar Pradesh 201002 India; 3 Structural Bioinformatics Lab, CSIR-Institute of Himalayan Bioresource Technology, Palampur, Himachal Pradesh 176061 India; 4 Chemical Technology Division, CSIR-Institute of Himalayan Bioresource Technology, Palampur, Himachal Pradesh 176061 India; 5 CSIR-Center for Cellular and Molecular Biology, Annexe-II, Medical Biotechnology Complex, Uppal Road, Hyderabad, Telangana 500007 India

**Keywords:** Protein analysis, Virtual drug screening, Biochemistry, Biotechnology, Computational biology and bioinformatics, Drug discovery

## Abstract

The main protease (M^pro^) of SARS-CoV-2 has been recognized as an attractive drug target because of its central role in viral replication. Our previous preliminary molecular docking studies showed that theaflavin 3-gallate (a natural bioactive molecule derived from theaflavin and found in high abundance in black tea) exhibited better docking scores than repurposed drugs (Atazanavir, Darunavir, Lopinavir). In this study, conventional and steered MD-simulations analyses revealed stronger interactions of theaflavin 3-gallate with the active site residues of M^pro^ than theaflavin and a standard molecule GC373 (a known inhibitor of M^pro^ and novel broad-spectrum anti-viral agent). Theaflavin 3-gallate inhibited M^pro^ protein of SARS-CoV-2 with an IC_50_ value of 18.48 ± 1.29 μM. Treatment of SARS-CoV-2 (Indian/a3i clade/2020 isolate) with 200 μM of theaflavin 3-gallate in vitro using Vero cells and quantifying viral transcripts demonstrated reduction of viral count by 75% (viral particles reduced from Log10^6.7^ to Log10^6.1^). Overall, our findings suggest that theaflavin 3-gallate effectively targets the M^pro^ thus limiting the replication of the SARS-CoV-2 virus in vitro.

## Introduction

The ongoing COVID-19 pandemic due to SARS-CoV-2 has paralyzed the whole world, motivating the scientific community worldwide to find possible remedies^1^. The COVID-19 outbreak in December 2019 developed into a global pandemic in just a few months, spreading to more than 222 countries, areas, or territories^2–4^. The SARS-CoV-2 belongs to the family of enveloped, single-stranded, positive-sense, and very diverse RNA viruses^1^. The genome of SARS-CoV-2 is composed of about 30,000 nucleotides: its replicase gene encodes for two overlapping polyproteins, namely pp1a and pp2ab, required for the replication and transcription of virus^4–6^. The polyproteins are proteolytically processed, mainly by the 33.8-kDa main protease (M^pro^), also known as 3C-like protease^7^. M^pro^ undergoes autolytic cleavage from polyproteins pp1a and pp1ab, which cleaves the polyprotein at 11 conserved sites^7^. Such functional importance of M^pro^ in the life cycle of the virus, along with the absence of its closely related homologs in human beings, recognize M^pro^ as an attractive target for the anti-viral drug designs^7^. Though several vaccines against SARS-CoV-2 have been developed and granted emergency use authorization by the Food and Drug Administration, there is still uncertainty about their long-term side effects, and no strong scientific data is available regarding the safety of these vaccines for pregnant or breastfeeding women^8^. Afshar et al.^9^ reported vaccination against SARS-CoV-2 in patients with co-morbidities and special groups people (e.g., cancer, organ transplant recipients, chronic liver diseases, diabetes mellitus, autoimmune disorders, end-stage renal disease, neurological disorders, chronic obstructive pulmonary disease, HIV, smokers, pregnant women, breastfeeding women, elderly people, children, patients with allergic reactions) need continuous medical monitoring post-immunization since the long-term effects of vaccination in these vulnerable groups are yet to be scientifically evidenced. A recent finding suggests that in the USA, up to one-third of the population is unsure of being vaccinated against the SARS-CoV-2 or is sure they will not do so^10^. Moreover, it is common to observe re-infections flaring up in immunized individuals as absolute immunity is not acquired post-immunization from an infection or vaccine^11–14^. Therefore, potential molecules that can inhibit viral replication and disrupt the interaction between the viral protein and host receptor can add significant value in managing COVID-19^11^. Several anti-viral compounds were screened *in-silico* in various laboratories worldwide to facilitate rapid drug discoveries. For example, Ghahremanpour et al. identified manidipine, boceprevir, lercanidipine, bedaquiline, and efonidipine as inhibitors of M^pro^ protein of SARS-CoV-2^15^. Similarly, 2-phenyl-1,2-benzoselenazol-3-one class of compounds were reported to inhibit M^pro^ protein of SARS-CoV-2 at nanomolar concentrations^16^. However, these studies still lack sufficient wet lab experimentations following clinical trials to support their claims. Many natural molecules and their derivatives have entered different stages of drug design, including clinical trials against diseases like cardiovascular diseases, malaria, HIV-AIDS, etc.^17–19^, and more than 50% of approved drugs are reported to be based on natural products^20^. Therefore, natural molecules have caught the attention of researchers in hunting for anti-SARS-CoV-2 drug candidates, and several natural molecules belonging to different classes and sources of origin have been explored through in silico and experimental investigations^21,22^. Natural molecules such as theaflavin, rutin, curcumin, salvianolic acid, and many flavones were reported to have anti-SARS-CoV-2 activity^21–25^. Among natural molecules, tea polyphenols are well known to exhibit anti-HIV, anti-cancer, anti-oxidative, anti-mutagenic, anti-diabetic, and hypocholesterolemic activities^26,27^. The beneficial effects of green tea, oolong tea, and black tea have been well-known since long ago^28^. Recent studies have also shown the anti-SARS-CoV-2 potential of several bioactive tea molecules. For example, epigallocatechin gallate was shown to disrupt the interaction of spike protein of SARS-CoV-2 with angiotensin-converting enzyme 2 receptors of host cells^29^. Epicatechin-3,5-di-O-gallate, epigallocatechin-3,5-di-O-gallate, and epigallocatechin-3,4-di-O-gallate were identified as inhibitors of RNA-dependent RNA polymerase^30^. Through *in-silico* studies, our group has previously recognized three lead molecules, oolonghomobisflavan-A, theasinensin-D, and theaflavin-3-O-gallate, as potential inhibitors of M^pro^ protein of SARS-CoV-2 with docking scores higher than repurposed drugs Atazanavir, Darunavir, and Lopinavir^30^. Since the average content of oolonghomobisflavan-A (1.1 mg/100 g) and theasinensin-D (1.8 mg/100 g) as compared to theaflavin 3-gallate (148.6 mg/100 g) in different types of tea is lower, we selected the later for detailed studies^31^. Additionally, oolonghomobisflavan-A and theasinensin-D are poorly soluble in water^32^, while theaflavin and its derivatives are highly soluble^33^. Considering the above-stated reasons and the enormous therapeutic potential of theaflavin 3-gallate^34,35^, we performed wet-lab studies to test the inhibition potential of theaflavin 3-gallate along with GC376 (known inhibitors of M^pro^) and theaflavin (precursor for theaflavin 3-gallate) against M^pro^ protein of SARS-CoV-2. The cell-based assay was performed to confirm the antiviral activity of theaflavin 3-gallate. Further, in silico studies were also conducted to shed insights into the mechanism of action of theaflavin 3-gallate on M^pro^.

## Results

### Theaflavin 3-gallate inhibited M^pro^ protein of SARS-CoV-2

**Figure 1:**
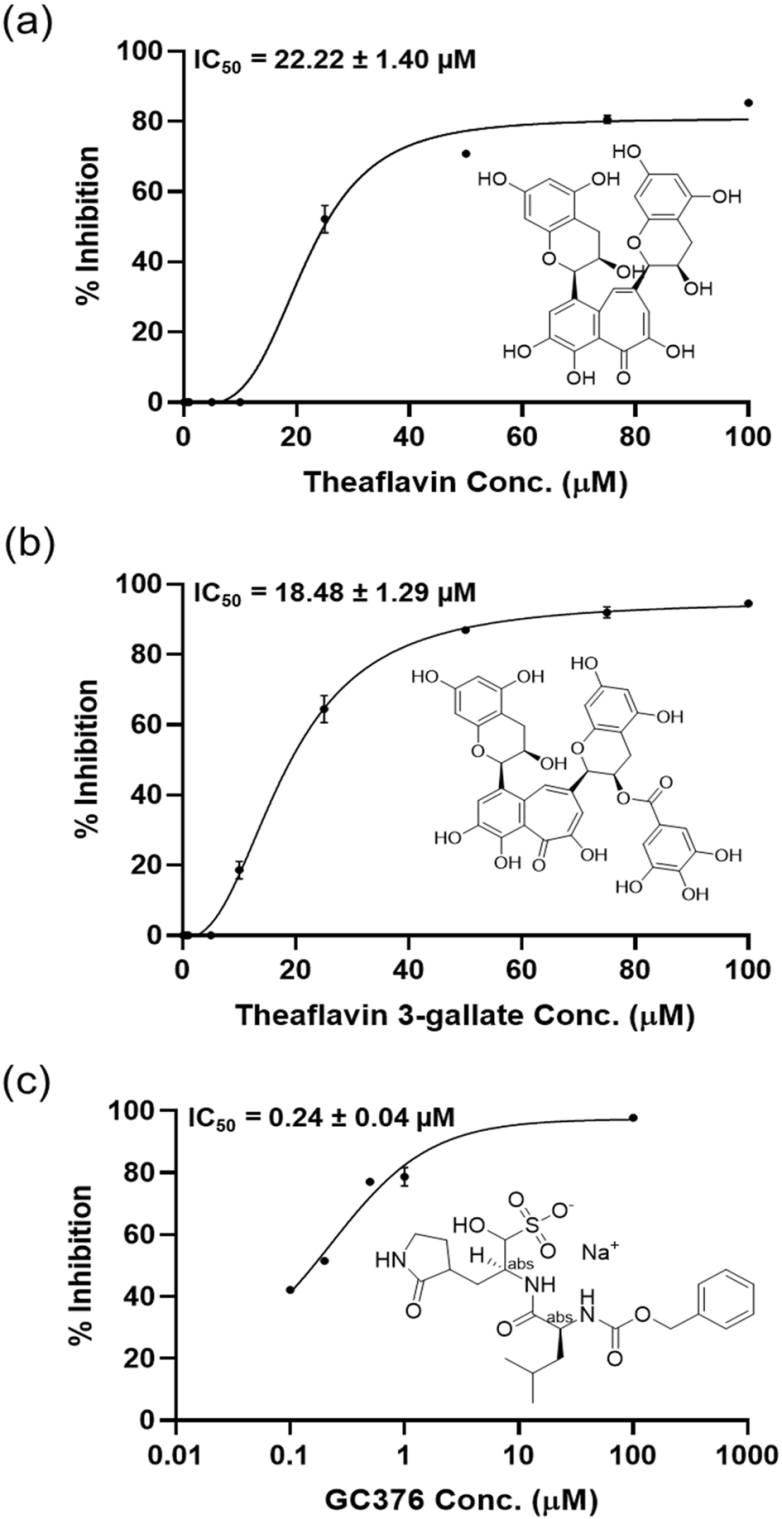
Inhibition of M^pro^ protein of SARS-CoV-2 by theaflavin 3-gallate. The inhibition of M^pro^ protein by (**a**) theaflavin (positive control), (**b**) theaflavin 3-gallate, and (**c**) GC376 (positive control) was measured in the presence of increasing concentrations of these molecules. The structures of molecules are shown along with the IC_50_ curves. Dose–response curves for IC_50_ values were determined by non-linear regression. Data represent mean ± SE, n = 3 independent replicates.

M^pro^ was inhibited by more than 80% after incubation with 100 µM of theaflavin and theaflavin 3-gallate for 30 min. Accordingly, it was tested for inhibition by these molecules at lower concentrations. The IC_50_ of theaflavin and theaflavin 3-gallate against M^pro^ protein was calculated to be 22.22 ± 1.4 μM and 18.48 ± 1.29 μM, respectively (Fig. 1). A known inhibitor of M^pro^ protein named GC376 was kept as a positive control. The IC_50_ for GC376 was calculated to be 0.24 ± 0.04 μM (Fig. 1) which is slightly lower than the reported value of 0.42 μM with the kit.

### Theaflavin 3-gallate reduced viral count in vitro in a dose-dependent manner

**Figure 2:**
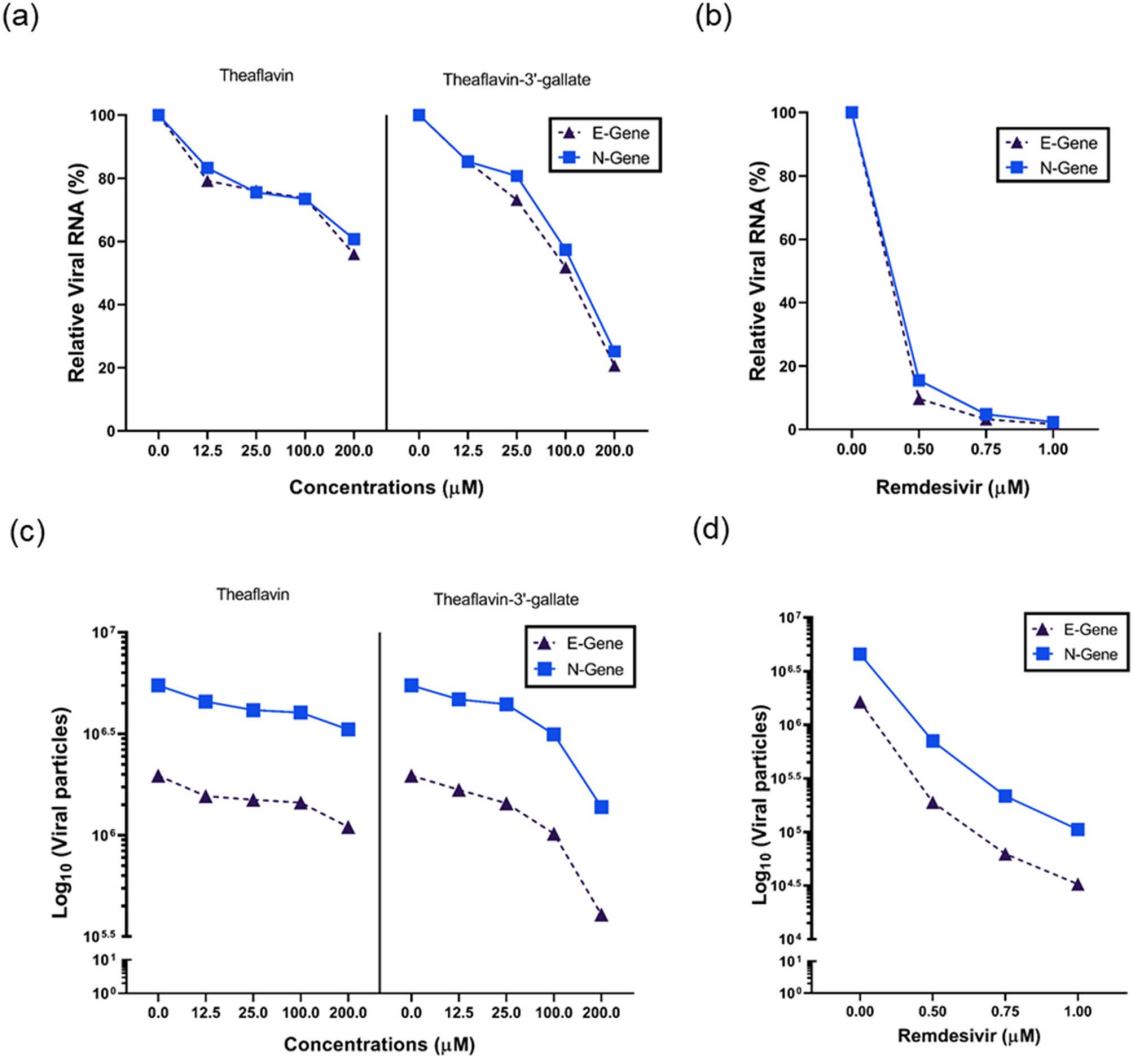
Effect of theaflavin 3-gallate on the inhibition of SARS-CoV-2. Response to theaflavin (positive control), theaflavin 3-gallate, and remdesivir (positive control) in Vero cells at 50, 100, 150, and 200 μM was calculated using quantitative PCR of N and E viral genes. Graphs represent relative viral RNA % (**a**,**b**) and log reduction in viral particles (**c**,**d**) after treatment with theaflavin, theaflavin 3-gallate, and positive control remdesivir, respectively.

Incubation of theaflavin and theaflavin 3-gallate with SARS-CoV-2 reduced the viral count to different extents (Fig. 2). It is important to emphasize that the observed decrease in the viral count was done by quantifying viral transcripts under in vitro conditions, i.e., experimental setup using Vero cells, not with a living organism. At lower concentrations of 12.50, 25 µM, theaflavin, and theaflavin 3-gallate could reduce the viral count to below 24%. However, the viral count was reduced by 26% with theaflavin and 42% with theaflavin 3-gallate at 100 µM. At 200 µM, theaflavin and theaflavin 3-gallate showed a reduction of 40% and 75%, respectively. Treatment with theaflavin 3-gallate at 200 µM reduced the viral particles from Log10^6.7^ to Log10^6.1^. As anticipated, treatment with remdesivir (positive control) resulted in the inhibition of the virus at a concentration of 0.5, 0.75, and 1.0 µM (Fig. 2).

### Theaflavin 3-gallate interacts with the active site residues of M^pro^

**Figure 3:**
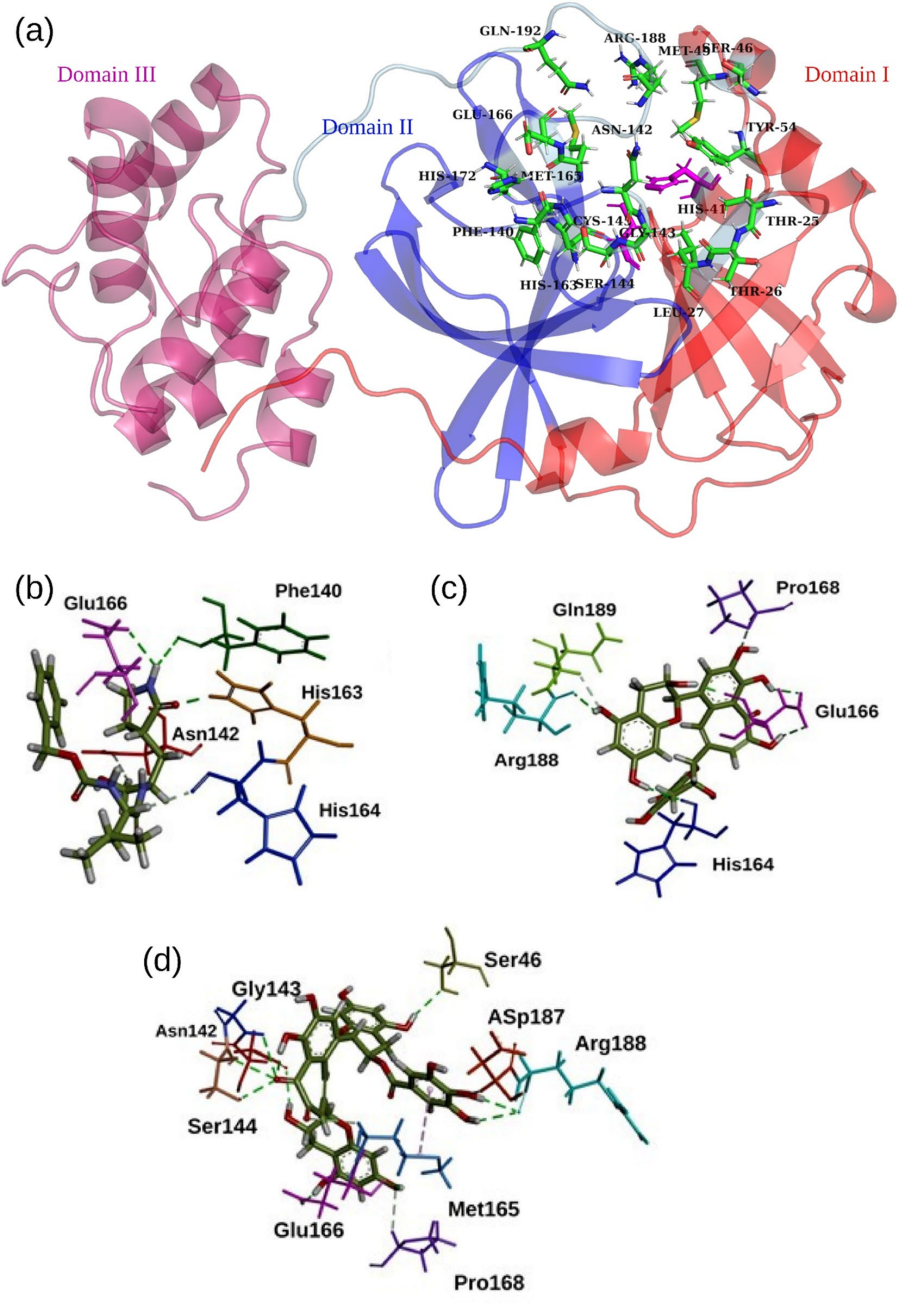
Analysis of docking results. (**a**) Different domains of M^pro^ and 3D representations of docking poses for (**b**) GC373, (**c**) theaflavin, and (**d**) theaflavin 3-gallate.

The availability of protein crystal structures allowed us to generate and visualize the interactions between protein and their respective ligands. The optimal poses of GC373 (the active form of the prodrug GC376), theaflavin, and theaflavin 3-gallate with the M^pro^ (PDB ID: 6M0K, resolution 1.50 Å) of SARS-CoV-2 were generated by employing molecular docking methodology. The docking scores in terms of interaction energy for GC373, theaflavin, and theaflavin 3-gallate were 50.54 kcal/mol, 57.41 kcal/ml, and 74.35 kcal/mol, respectively. The binding site on M^pro^ is present between domains I and II, as shown in Fig. 3a. The best poses with the highest docking score were selected, as shown in Fig. 3b–d. The 2D interaction poses are shown in Fig. S1. The standard inhibitor (GC373) formed three conventional hydrogen bonds (H-bonds), two carbon-hydrogen bonds, and pi-pi interactions with the active site of M^pro^. Theaflavin showed a higher number of H-bonds than the standard molecule. A total of eight H-bonds were observed between theaflavin and the binding pocket residues of M^pro^. The residue Glu166 interacted via three H-bonds, while the residues His41, His164, Pro168, Arg188, and Gln189 formed H-bond each. Among the docked molecules, theaflavin 3-gallate showed the highest number of H-bonds. Theaflavin 3-gallate established eleven H-bonds with residues of the active site of SARS-CoV-2 M^pro^. The residues involved in H-bonds with theaflavin 3-gallate were Ser46, Ser144, Asn142, Gly143, Met165, Glu166, Pro168, Asp187, and Arg188. The pi-pi interactions were shown by residues Met49 and Cys145 of the binding pocket. All three molecules interacted with the key residues of the binding site. Many natural molecules were shown to target the same binding site to inhibit the M^pro^ of SARS-CoV-2. The stability of binding poses of theaflavin 3-gallate with M^pro^ was further accessed by performing MD simulations.

### MD simulation revealed structural stability of the M^pro^-ligand complexes

**Figure 4:**
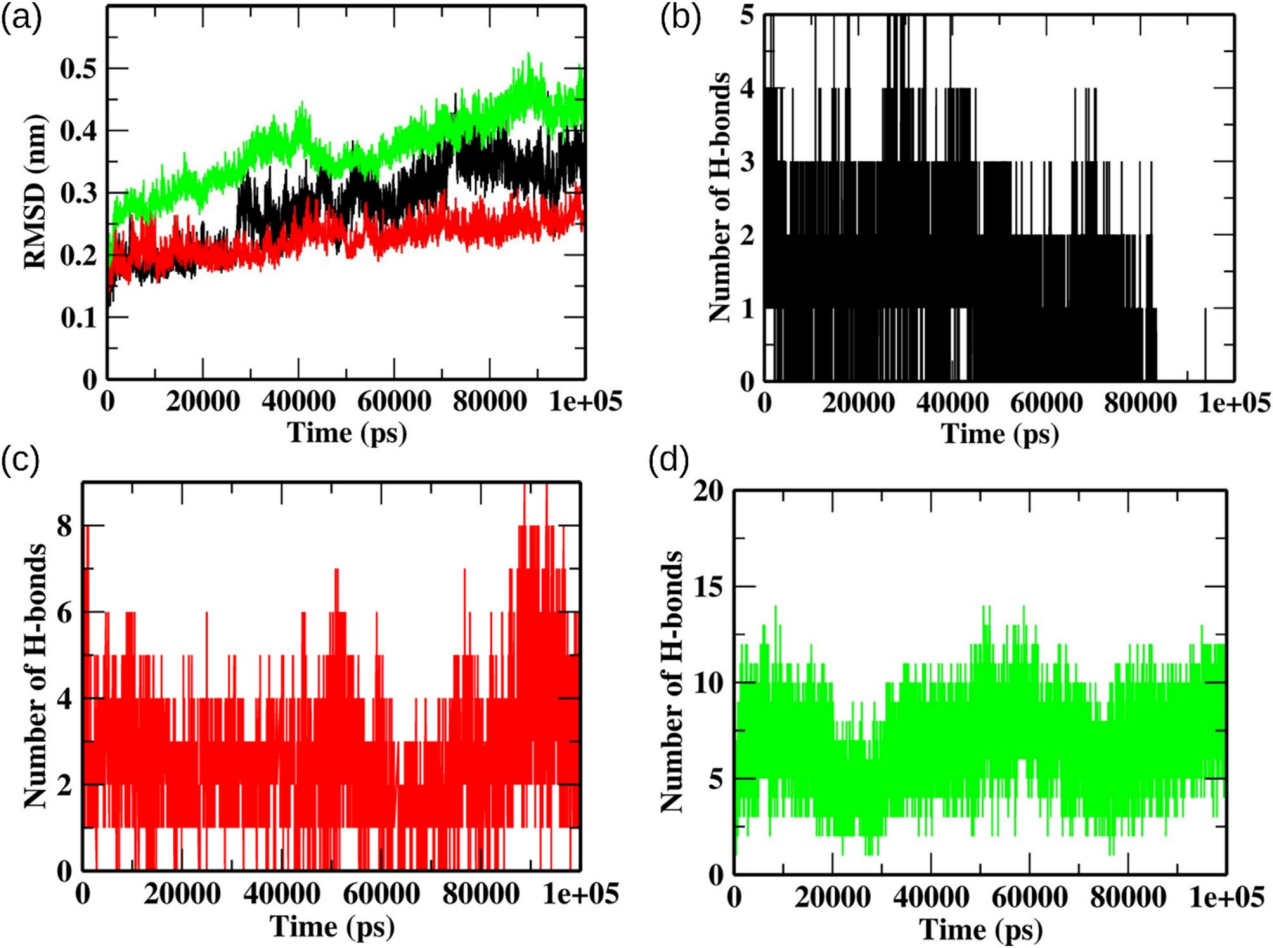
Analysis of MD trajectories. (**a**) RMSD of backbone Cα atoms and (**b**–**d**) number of H-bonds formed between M^pro^ and ligands during the entire simulation. The color-coding scheme is as follows: GC373 (black), theaflavin (red), and theaflavin 3-gallate (green).

MD simulations present various analyses to understand the dynamics involved in protein–ligand interactions at the molecular level. We calculated the root mean square deviation (RMSD) of backbone C-α atoms to investigate the effect of ligand binding on protein topology (Fig. 4a). We observed initial deviations from the starting structures during the simulation in RMSD values for all three trajectories. The RMSD values for protein structure with theaflavin stabilized at ~ 10 ns and followed the same trajectory till the end of the simulation. On the other hand, the RMSD values of protein with GC373 and theaflavin 3-gallate stabilized towards the end of the simulation. However, the simulation's average RMSD values never exceeded over 0.4 nm. The average RMSD values for the entire simulation run for GC373, theaflavin, and theaflavin 3-gallate were 0.27 nm, 0.22 nm, and 0.36 nm, respectively. The low RMSD values and stable trajectories validated the structural stability and indicated that the ligand-binding had no impact on the overall protein topology.

### Theaflavin 3-gallate formed H-bonds and exhibited some exclusive interactions with the binding site of M^pro^

We analyzed the total number of H-bonds formed between M^pro^ and selected molecules during the simulation (Fig. 4b–d). Among the three molecules, theaflavin 3-gallate formed the highest number of H-bonds throughout the entire simulation run. The average H-bonds for GC373, theaflavin, and theaflavin 3-gallate were 2.15, 3.26, and 6.85, respectively. We observed no H-bonds between GC373 and M^pro^ after 80 ns of simulation time. A few conformations of the M^pro^-theaflavin 3-gallate complex showed up to 13 H-bonds during the simulation. These results suggest a high potential of theaflavin 3-gallate over theaflavin and GC373 to interact with the active site of M^pro^.

The MD trajectories could be used to visualize the interactions between protein and ligands at different time intervals during the simulation. We also took snapshots of protein–ligand complexes at different time intervals to analyze the interaction pattern of GC373 (Fig. S2), theaflavin (Fig. S3), and theaflavin 3-gallate (Fig. S4) with the binding sites of M^pro^. The standard molecule GC373 initially interacted by showing H-bonds, pi-pi, and hydrophobic interactions, while towards the end of the simulation, it only interacted with the active site residues via hydrophobic interactions. The binding site of M^pro^ formed the greatest number of H-bonds with theaflavin 3-gallate in all the conformations when compared to GC373 and theaflavin. We also observed the formation of some exclusive interactions (Val186, His164, His41) at different time intervals between M^pro^ and theaflavin 3-gallate during the simulation. These interactions were not observed for GC373 and theaflavin. Also, theaflavin formed weaker hydrophobic interactions with residue Cys145, while theaflavin 3-gallate interacted with Cys145 by stronger H-bonds, pi-sulfur, and pi-alkyl interactions. The interacting residues and the interactions between M^pro^ and theaflavin 3-gallate at different time intervals are shown in Table 1. These results confirmed that theaflavin 3-gallate adhered tightly to the binding site by interacting with different residues throughout the simulation.

**Table 1:** The interacting residues between Theaflavin 3-gallate and M^pro^ at different time intervals during the simulation.

Time	Conventional hydrogen bonds	Pi-alkyl	Pi-Pi T-shaped	Pi-sulfur	Van der Waals
20 ns	His41, Cys44, Gly143, Ser144, Glu166, Val186, Asp187, Gln189,	Leu27	–	Cys145, Met165	Thr25, Thr26, Thr45, Ser46, Met49, Asn142, Leu167, Pro168, Asp178, Phe181,Asp187, Arg188, Ala191, Gln192,
40 ns	His41, Cys44, Ser46, Asn142, Ser144, Cys145, Val186, Arg188, Gln189, Gln192	Met165	His41	Cys145	Thr25, Thr26, Leu27, Thr45, Phe81, Gly143, Arg188
60 ns	His41, Cys44, Asn142, Ser144, Cys145, His164, Glu166, Val186, Asp187, Gln189, Thr190, Ala191	Met165	–	Cys145, Met165	Thr25, Thr26, Leu27, Asn28, Thr45, Leu50, Asn119, Gly143, Phe181, Asp187, Thr190, Ala191
80 ns	His41, Ser46, Ala91 Val186, Gln189,	Cys145, Ala191	Phe185	Met165	Thr25, Leu27, Val42, Cys44, Thr45, Gly143, his164, Phe181, Asp187

### Van der Waal and electrostatic energies contributed to the tight binding of theaflavin 3-gallate to M^pro^

The Molecular Mechanics Poisson-Boltzmann Surface Area (MMPBSA) is a well-established end-state post-processing approach for calculating the ligands' free energy binding to their respective receptors. The total binding energy obtained by the MMPBSA approach is a cumulative sum of individual energies, including the polar solvation energy, van der Waal energy, SASA energy, and electrostatic energy. The MMPBSA results for the three simulation blocks (0–100 ns, 40–50 ns, and 90–100 ns) are summarized in Table 2. Among the selected molecules, theaflavin 3-gallate showed the least binding energy (highest affinity) of − 48.02 ± 1.61 kcal/mol, followed by theaflavin and GC373. The van der Waal energy contributed most significantly to the binding between ligands and M^pro^ of SARS-CoV-2. The non-favorable contributions by the polar solvation energy were highest for theaflavin 3-gallate. However, the large difference in positive contributions by van der Waal and electrostatic energy contributed to the least binding energy of theaflavin 3-gallate compared to GC373 and theaflavin. The final binding energy of theaflavin 3-gallate provided a strong rationale for its high inhibition potential against both the proteins. The per-frame trajectories of binding energies for all the three complexes throughout the simulation are provided in Fig. S5.

**Table 2:** The average values of components of the binding free energy calculated by the MMPBSA method over three simulation blocks (0–10 ns, 40–50 ns, and 90–100 ns).

Complex	Van der Waal energy (kcal/mol)	Electrostatic energy (kcal/mol)	Polar solvation energy (kcal/mol)	SASA energy (kcal/mol)	Binding energy (kcal/mol)
GC373	− 22.20 ± 1.49	− 7.56 ± 0.88	25.26 ± 2.51	−﻿ 5.01 ± 0.44	− 9.52 ± 1.41
Theaflavin	− 31.86 ± 3.67	− 14.51 ± 1.71	40.91 ± 1.15	− 5.92 ± 0.19	− 11.21 ± 1.66
Theaflavin 3-gallate	− 63.42 ± 1.2	− 15.66 ± 1.64	41.85 ± 1.40	− 5.90 ± 0.46	− 48.02 ± 1.61

### A strong external pulling force is required to unbind theaflavin 3-gallate from M^pro^

**Figure 5:**
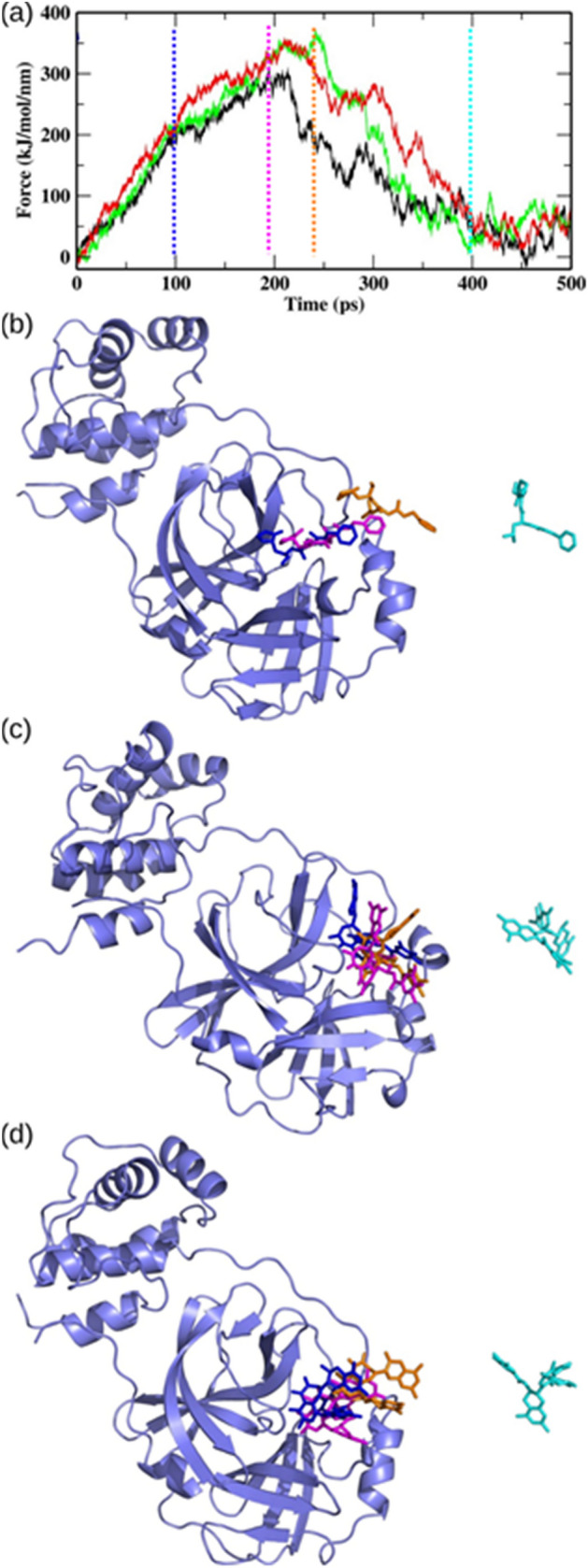
Analysis of SMD results showing: (**a**) typical external force profiles of GC373 (black), theaflavin (red), and theaflavin 3-gallate (green); the position of (**b**) GC373, (**c**) theaflavin, and (**d**) theaflavin 3-gallate at different time intervals during SMD simulations. The color-coding is as follows: 100 ns (blue), 194 ns (magenta), 242 ns (orange), and 400 ns (cyan).

SMD simulations can offer the qualitative perspectives of interactions and conformational perturbations between ligand and its neighboring amino acids by inducing ligand unbinding along the MD simulation pathway. In this study, the well equilibrated protein–ligand complexes were taken as starting points for SMD simulations. A pulling simulation was performed on all the complexes with a constant pulling velocity of 0.01 nm/ps and a spring constant of 250 kJ/mol/nm^2^. The representative force profiles of GC373, theaflavin, theaflavin 3-gallate unbinding from binding sites of the M^pro^ are shown in Fig. 5. All the complexes observed a linear increase in the time-dependent external pulling force for the initial phase of SMD simulation. The standard molecule GC373 required the lowest amount of external pulling force to completely unbind from the binding pocket. The peak values of external pulling force experienced by theaflavin and theaflavin 3-gallate were ~ 350.02 kJ/mol/nm and ~ 368.47 kJ/mol/nm, respectively. Afterward, the external pulling force gradually decreased, and the ligand was entirely out of the binding site. The time taken by theaflavin 3-gallate to achieve the maximum pulling force was greater than theaflavin and GC373, suggesting that theaflavin 3-gallate was bound to the active site for a longer duration than both the compounds. These results show that theaflavin 3-gallate required a strong external pulling force to separate it from the binding site of M^pro^ of SARS-CoV-2.

## Discussion and conclusions

The emergence of highly pathogenic human viruses, SARS-CoV and MERS belonging to the β-coronavirus group, has gained attention due to their zoonotic transmission and became major pathogenic strains^36,37^. The timely development of anti-viral drugs is of utmost importance. Still, drug development in a short span is exceptionally challenging due to inadequate knowledge of the zoonotic source. Drug repurposing could be an alternative in which the molecules/compounds of known therapeutic effects can be screened and tested for inhibition of viruses, including SARS-CoV-2^38^. Few plant-based compounds offer a rich reservoir for novel anti-viral drug development. They have been reported to possess anti-viral activity against Herpes Simplex Virus type-1, Coronavirus, Influenza Virus, Hepatitis C virus, and Human Immunodeficiency Virus^39–41^. Our previous *in-silico* studies showed that the active molecule theaflavin 3-O-gallate has a strong affinity to the substrate-binding pocket of M^pro^ of SARS-CoV-2^42^. To validate this observation, we quantified the inhibitory potential (IC_50_) of theaflavin 3-gallate against M^pro^ protein of SARS-CoV-2, which was found to be 18.48 ± 1.29 μM (Fig. 1). Also, incubation of theaflavin 3-gallate with SARS-CoV-2 virus exhibited a 75% reduction of the viral count at a concentration of 200 μM (Fig. 2).

To understand the mechanism of inhibitory action of theaflavin 3-gallate, we performed molecular docking studies, which provide a platform to predict the estimated binding affinity and optimal binding pose between receptor and ligand. The binding poses of theaflavin 3-gallate were compared with a standard drug GC373 and parent molecule theaflavin. Molecular docking analysis showed that theaflavin 3-gallate interacts strongly with the binding sites on M^pro^ with a higher docking score than GC373 and theaflavin. Theaflavin 3-gallate acquired the active site of M^pro^ by interacting with many residues (Asn142, Ser144, His145, His163, and Glu166) (Fig. 3), crucial for dimerization and biological activity. Many experimental and computational studies have shown potent inhibitor molecules interacting with these residues^7,23,24,30,43,44^. The stability of binding poses was validated by different MD-driven time-dependent analyses (Fig. 4). The low deviations in RMSD values for all the three structures (Fig. 4) suggested that the binding of ligands on protein had no impact on the stability of the binding pocket. We also compared the number of H-bonds formed by GC373, theaflavin, and theaflavin 3-gallate with the binding site of M^pro^ throughout the simulation. The analysis of the H-bond profiles of the three molecules showed that theaflavin 3-gallate formed the highest number of bonds during the simulation (Fig. 4d). Protein–ligand conformations at different time intervals were extracted from the MD trajectories to get an in-depth insight into the molecular interactions during the simulations. Among the three compounds, theaflavin 3-gallate formed exclusive H-bonds with residues His41, Val186, and His164 during the simulation, whereas these interactions were absent in theaflavin and GC373 (Fig. 4b,c).

Theaflavin 3-gallate strongly interacts with Cys145 residue involved in the formation of the catalytic dyad of the active site of M^pro^^7^ compared to theaflavin and GC373. The strong interactions shown by theaflavin 3-gallate with M^pro^ were further validated and compared with GC373 and theaflavin by calculating the binding free energy by the MMPBSA method, which is an efficient and reliable method for evaluating protein–ligand interactions^45,46^. The binding free energy results confirmed that theaflavin 3-gallate showed the highest binding affinity for M^pro^ than GC373 and theaflavin (Table 2). Our results showed that the van der Waal energy contributed most favorably to the binding of theaflavin 3-gallate with M^pro^ (Table 2). Moreover, we also performed SMD simulations to analyze the amount of external force required for unbinding GC373, theaflavin, and theaflavin 3-gallate from the binding pocket. Our SMD results demonstrated that theaflavin 3-gallate required the highest amount of external force to unbind it from the binding pocket of the M^pro^ of SARS-CoV-2 (Fig. 5). Also, theaflavin 3-gallate remained at the active site for longer than the GC373 and theaflavin during the pulling simulations (Fig. 5), suggesting strong interactions with the residues of the binding site.

In conclusion, theaflavin 3-gallate exhibited a strong binding affinity with M^pro^ as evidenced by in-silico, and enzyme-based inhibition studies and reduced SARS-CoV-2 virus count as evident from cell-based experimental results. The overall results suggest the potential use of theaflavin 3-gallate against SARS-CoV-2. Theaflavin 3-gallate is a major component of black tea which is already known for its anti-oxidant properties and is the most consumed beverage in the world. Since theaflavin 3-gallate is already consumed by humans through tea and crosses cell–cell barriers in the body^47^, it is an excellent potential inhibitor against SARS-CoV-2. Either the molecule alone or in formulations with other such anti-viral compounds as a cocktail can provide an effective first line of defense against diseases associated with coronaviruses.

## Methods

Theaflavin (molecular weight = 564.49 g/mol) and theaflavin 3-gallate (Molecular weight = 716.61 g/mol) were procured from (PhytoLab GmbH & Co. KG, Germany). The authenticity and purity of the molecules were validated by mass spectrometry analysis using UHPLC-IM-QTOF 6560 instrument (Agilent, USA) equipped with a PDA detector and hyphenated to the Q-TOF MS/MS (Fig. S6). Stock solutions (5 mM) of theaflavin and theaflavin 3-gallate were prepared in methanol. Appropriate dilutions were made in assay buffer to test the molecules at various concentrations keeping methanol concentration less than 1% in the final reaction volume.

All the assays were carried out at room temperature (RT: 25 ± 2 °C) in triplicates. As an initial screen, theaflavin and theaflavin 3-gallate were tested for inhibition of M^pro^ protein at 100 µM concentration and later tested at lower concentrations to calculate the IC_50_ value.

### In vitro inhibition assay of M^pro^

MBP-tagged (SARS-CoV-2) assay kit was used (Catalogue No. #79955-1; BPS Bioscience, USA) to study the inhibition of M^pro^ by theaflavin 3-gallate. GC376 and theaflavin (known inhibitors of M^pro^) were kept as positive controls. In brief, reactions were performed in a final assay volume of 50 µl in a 96 well plate wherein each reaction consisted of 30 µl of M^pro^ (150 ng) prepared in an assay buffer containing 1 mM DTT, 10 μl of fluorogenic protease substrate (DABCYL-KTSAVLQSGFRKME-EDANS) to a final concentration of 50 μM, and 10 µl of either theaflavin or theaflavin 3-gallate. Appropriate assay control was also kept by replacing inhibitor with inhibitor buffer. Sample controls were included to account for any interference caused by the color of the molecules. The plate was sealed with the plate sealer and incubated overnight at RT. The fluorescence intensity was measured in a microtiter plate-reading fluorimeter (Biotek Synergy™ H1, USA) with an excitation wavelength of 360 nm and detection of emission at 460 nm. All the values were corrected by subtracting the blank values. The percent inhibition was calculated using the following formula: % inhibition = [(positive control − test inhibitor)/positive control] × 100.

### Calculation of IC_50_

Theaflavin and theaflavin 3-gallate were tested at concentrations of 0.1, 0.6, 1.0, 5.0, 10, 25, 50, 75, and 100 μM against M^pro^ protein of SARS-CoV-2 to calculate inhibitory concentration (IC_50_) values. GC376 was tested at 0.1, 0.2, 0.5, 1, and 100 μM. The inhibitor concentration was plotted against the percent inhibition, and the IC_50_ value was calculated using the non-linear regression equation of the resulting graph using GraphPad Prism software version 8.0.2.

### Drug treatment and detection of SARS-CoV-2 using an RT-qPCR assay

The individual anti-viral effect of theaflavin and theaflavin 3-gallate were tested against the SARS-CoV-2 (Indian/a3i clade/2020 isolate) virus in vitro using Vero cells (Green African Monkey). The Vero cells, prior to infection, were maintained in Dulbecco Minimum Essential Medium (DMEM, Gibco) with 10% Fetal Bovine Serum (Gibco) at 37 °C, 5% CO_2_. Twenty-four hours prior to infection, the cells were trypsinized and seeded in a 96-well plate. 80–90% of cell confluency was considered for infection. Briefly, 50, 100, 150 and 200 (µM) concentrations of theaflavin and theaflavin 3-gallate were used to enumerate the anti-viral effect. Initially, the cells were primed with different concentrations of theaflavin and theaflavin 3-gallate for 2 h. Later, the medium containing the drug candidates was replaced with the virus inoculum (0.1 MOI) along with drug dilutions made in DMEM without FBS for 3 h. Post-infection, the media was replaced with DMEM media along with 10% FBS-containing drugs and maintained in an incubator at 37 °C, 5% CO_2_ until 72 h. Post 72 h, the cell supernatants were collected to enumerate the cell-released viral RNA particles using quantitative real-time PCR.

Viral RNA extraction was performed via the Viral/Pathogen Extraction Kit (Applied Biosystems, Thermo Fisher Scientific) according to the protocol described by the manufacturer. Viral supernatants (200µL) after centrifugation from the experimental groups were aliquoted into deep well plates and were subjected to the lysis buffer that contained 260 µL, binding solution; 10 µL, binding beads; 5 µL Proteinase K (w.r.t. to mentioned sample volume) from the extraction kit. The RNA extraction step was performed using the Kingfisher Flex (version 1.01, Thermo Fisher Scientific) according to the manufacturer’s protocol. The obtained RNA was stored at − 80 °C until further use. The COVID-19 RT-PCR Detection Kit (Fosun 2019-nCoV qPCR, Shanghai Fosun Long March Medical Science Co. Ltd.) was used. The primers provided along with the kit amplify the Envelope gene (E; ROX labeled), Nucleocapsid gene (N-JOE labeled), and Open Reading Frame1ab (ORF1ab, FAM-labeled) specific to SARS-CoV-2. SARS-CoV-2 cDNA showing a Ct ~ 28 was used as an assay control. A linear regression equation, obtained from the known log viral particle count and Ct using RT-qPCR, was established for N- and ORF1ab (via the COVID-19 RT-PCR Detection Kit) genes specific to SARS-CoV-2^44^.

### Molecular docking

We used the resolved crystal structure (resolution 1.50 Å) of M^pro^ (PDB ID: 6M0K)^38^ protein of SARS-CoV-2 for a molecular docking study through the CDOCKER protocol of Discovery Studio^48^. The structure of theaflavin and theaflavin 3-gallate were downloaded in SDF format from PubChem (CID: 169167)^49^. The Gaussian protocol with density function theory was used for ligand geometry and energy minimization^50^. The binding site for M^pro^ was defined by taking the reference of the co-crystallized inhibitor (11b), and the one with the best docking score was reported. The sphere coordinates for the M^pro^ binding pocket were 11.62, 11.88, and 68.70, with a radius of 12.00 Å. The other parameters were kept as default. The top five binding poses with the highest CDOCKER energy were reported of all the binding conformations predicted.

### Molecular dynamics (MD) simulations and thermodynamic free energy calculations

The best binding poses of theaflavin 3-gallate with M^pro^ were subjected to MD simulations by GROMACS package^51–53^. We used the CHARMM36 force field to generate ligand topologies by CGenFF server^54,55^. Similarly, the protein topologies were prepared by the CHARMM36 force field by employing the “gmx pdb2gmx” script of GROMACS. The protein–ligand complexes for simulations were prepared by appending ligand topologies to the protein topologies. To maintain an overall neutral charge of the system, sodium and chloride ions were added by employing the “gmx genion” script. Further steps of MD simulations were carried out by following the protocol defined in our previous studies^42,56,57^. The energy minimization of the simulated system was performed using the steepest descending algorithm for about 50,000 steps. After that, the system was subjected to the equilibration step using NVT for 1000 ps, followed by NPT ensembles for 1000 ps. Parrinello-Rahman pressure and Berendsen temperature controller systems were employed throughout the equilibration to maintain a constant pressure of 1 bar and temperature of 300 K, respectively. Finally, an MD production run of about 100 ns was employed without restrictions. The most frequently used particle mesh Ewald (PME) method was taken into account for calculating long-range electrostatic interactions. The Lennard–Jones potential with a cutoff value of 1 nm was utilized to calculate short-range van der Waals interactions. At the same time, Linear Constraint Algorithm (LINCS) was employed to constrain all the covalent bond lengths, including the hydrogen bond. The simulation trajectories of both the complexes were used to determine the root mean square deviations (RMSD) of backbone C-α atoms, extracting binding poses at different time intervals, and calculating thermodynamic binding free energies. We utilized the Molecular Mechanics Poisson-Boltzmann Surface Area (MMPBSA) method to calculate the free energies of protein–ligand binding. MM-PBSA is a classical and validated method for modeling protein–ligand interactions^46^.

### Steered molecular dynamics (SMD) simulations

SMD allows analyzing the amount of external force required to unbind ligand from its binding site in protein^58^. One of the most extensively used GROMACS packages was employed to perform SMD simulations. The end terminals of proteins were processed for preparing protein topologies, while the same ligand topologies were used in conventional MD simulations. Subsequently, protein–ligand complexes were solvated in a rectangular box of dimensions (8.5 × 8.3 × 25) Å^3^. The steepest descent method was employed for the energy minimization of protein–ligand complexes. Further, an NPT run was carried out at 100 ps to equilibrate the energy-minimized complexes. For the implementation of the pull code, a spring constant of 250 kJ/mol/nm^2^ and a constant velocity of 0.01 nm/ps was maintained. For the execution of the pulling code, a spring constant of 250 kJ/mol/nm^2^ and a constant velocity of 0.01 nm/ps was maintained. During pulling simulations, the following equation was utilized to calculate the external force:

$${\text{F}} = - {\text{k }}\left[ {{\text{X}}_{{{\text{pull}}}} \left( {\text{t}} \right){-}{\text{X}}_{{{\text{pull}}}} \left( 0 \right) - {\text{vt}}} \right]$$

where F = external pulling force; k = spring constant; v = constant velocity; X_pull_ (t) = position of atom at time t and X_pull_ (0) = initial position.

## Supplementary Information

Supplementary Information.

## Data Availability

The datasets used and/or analysed during the current study available from the corresponding author on reasonable request.
