# Supplementary material for: Theaflavin 3-gallate inhibits the main protease (Mpro) of SARS-CoV-2 and reduces its count in vitro
Source: Sci Rep. 2022 Jul 30;12:13146. doi: 10.1038/s41598-022-17558-5 (PMC9338964; doi:10.1038/s41598-022-17558-5)
Supplement: Supplementary file 1 — Supplementary Information. [file 41598_2022_17558_MOESM1_ESM.doc]

**Electronic Supplementary Information**

**Theaflavin 3-gallate inhibits the main protease (M^pro^) of SARS-CoV-2 and reduces its count *in-vitro***

Mahima Chauhan^1,2#^, Vijay Kumar Bhardwaj^1,2,3#^, Asheesh Kumar^1,2^, Vinod Kumar^1^, Pawan Kumar^2,4^, M. Ghalib Enayathullah^5^, Jessie Thomas^5^, Joel George^5^, Bokara Kiran Kumar^5*^, Rituraj Purohit^1,2,3*^, Arun Kumar^1,2*^, Sanjay Kumar^1^

Affiliations:

^1^Biotechnology Division, CSIR-Institute of Himalayan Bioresource Technology, Palampur, Himachal Pradesh, 176061, India

^2^Academy of Scientific and Innovative Research, Ghaziabad, 201002, Uttar Pradesh, India

^3^Structural Bioinformatics Lab, CSIR-Institute of Himalayan Bioresource Technology, Palampur, Himachal Pradesh, 176061, India

^4^Chemical Technology Division, CSIR-Institute of Himalayan Bioresource Technology, Palampur, Himachal Pradesh, 176061, India

^5^CSIR-Center for Cellular and Molecular Biology, Annexe-II, Medical Biotechnology Complex, Uppal Road, Hyderabad, Telangana, India, 500007

^#^Equal contribution

Corresponding authors*:

Dr. B Kiran Kumar: bokarakiran@ccmb.res.in

Dr. Rituraj Purohit: rituraj@ihbt.res.in

Dr. Arun Kumar: arunkumar@ihbt.res.in

**Methodology**

**LC-MS analysis**

UHPLC-IM-QTOF 6560 instrument (Agilent, USA) equipped with a PDA detector and hyphenated to the Q-TOF MS/MS was used to confirm the identity and purity of theaflavin and theaflavin 3-gallate. Following protocol was used for the analysis: the sample was dissolved in LC-MS grade methanol to make a final concentration of 1 mg/ml and filtered with a 0.22 µm PTFE syringe filter before injecting into the LC-MS system. LC conditions were as follows: The mobile phase used was 0.1% formic acid in water (channel A) and 0.1% formic acid in acetonitrile (channel B). Waters BEH-shield C18 column (2.1 mm × 100 mm and particle size of 1.7 µm) was used, and the column temperature was kept at 30 °C. One µl of sample solution was injected with the following gradient elution program (total run time of 15 min): Initial to 0.3 min, 5% B; 0.3 to 4 min, 5 to 55% B; 4 to 9 min, 55-75% B; 9-10 min, 75-90% B; 10-10.5 min, 90-90% B; 10.5-11 min, 90-5% B (Initial); and 11-15 min initial conditioning. Steep gradient with a flow rate of 0.25 ml/min was used throughout the method. MS conditions: source gas temperature, 300 °C; sealth gas temperature, 350 °C, sealth gas flow, 11 L/min; and cone gas flow, 5 L/min. Scan source parameters: capillary voltage, 3500 V; nozzle voltage; 1000 V; and fragmentor voltage, 400 V.

**Results**

The chromatograms of theaflavin and theaflavin 3-gallate at 375 nm wavelength showed peaks at an RT of 5.92 min and 6.053 min [Fig. S6(1,2)]. The UV absorbance peak pattern for theaflavin and theaflavin 3-gallate was of nearly similar peaks at wavelengths of 203 nm, 271 nm, 375 nm, and 459 nm [Fig. S6(1a, 2a)]. The m/z values of theaflavin and theaflavin 3-gallate were 565.1330 and 717.14, respectively [Fig. S6(1b, 2b)].


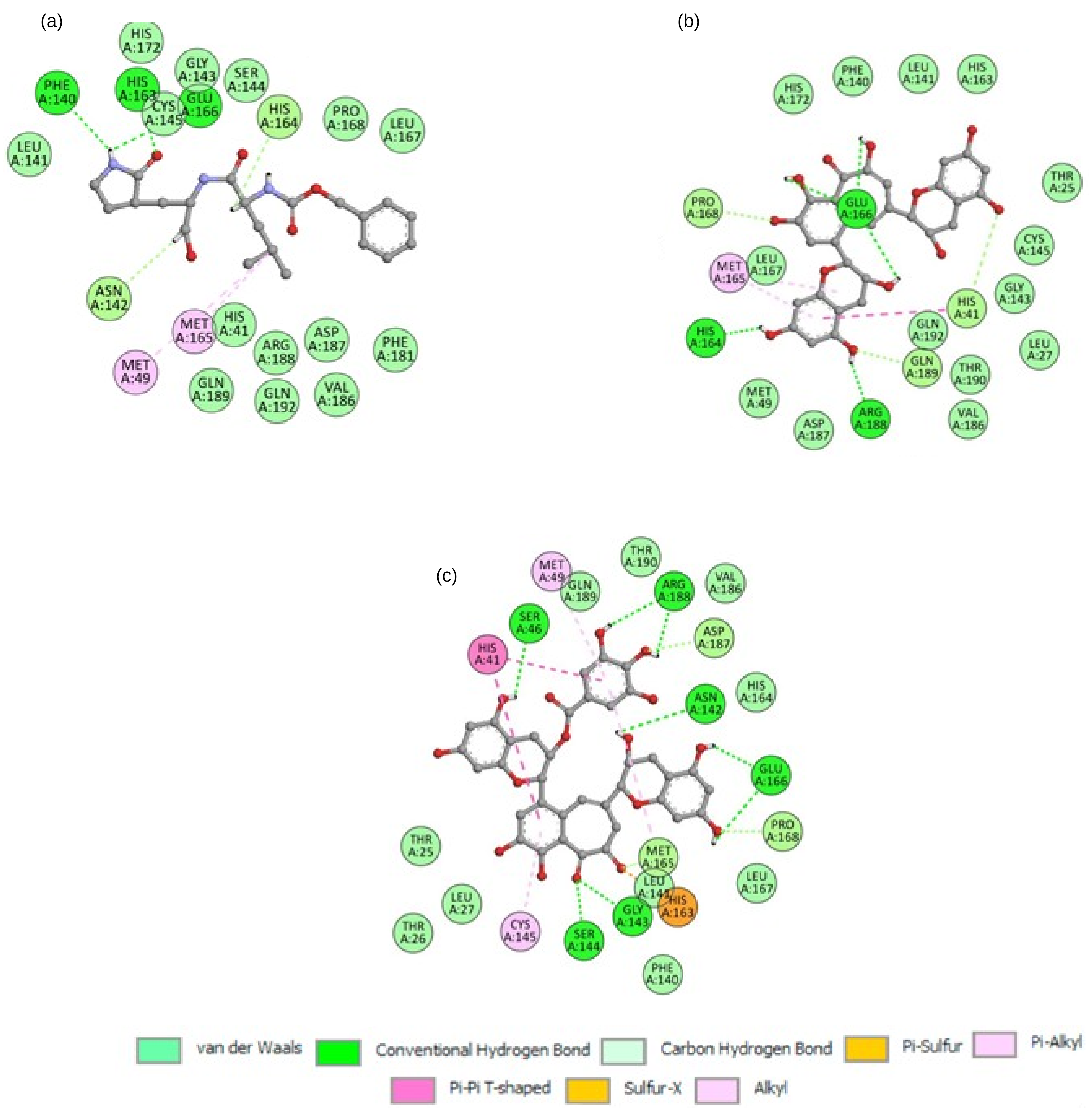


**Supplementary Fig. S1.** **Analysis of docking poses.** 2D interaction poses of M^pro^ with (a) GC373, (b) theaflavin, and (c) theaflavin 3-gallate.

**
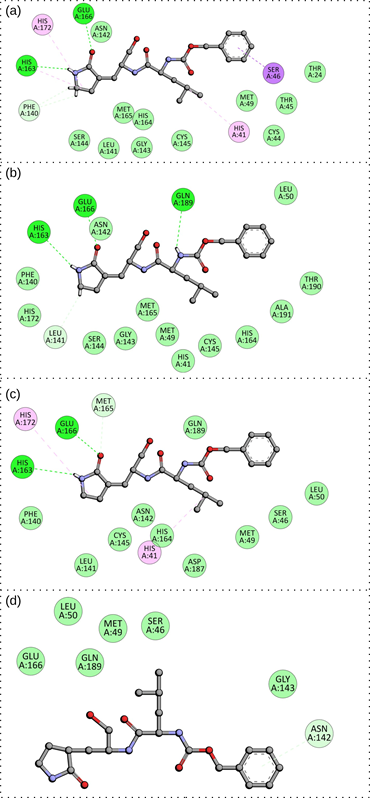
**

**Supplementary Fig. S2. Representation of the molecular interactions at the binding site of GC373-M^pro^ complex.** The binding poses are shown for time intervals (a) 20 ns, (b) 40 ns, (c) 60 ns, and (d) 80 ns.

**
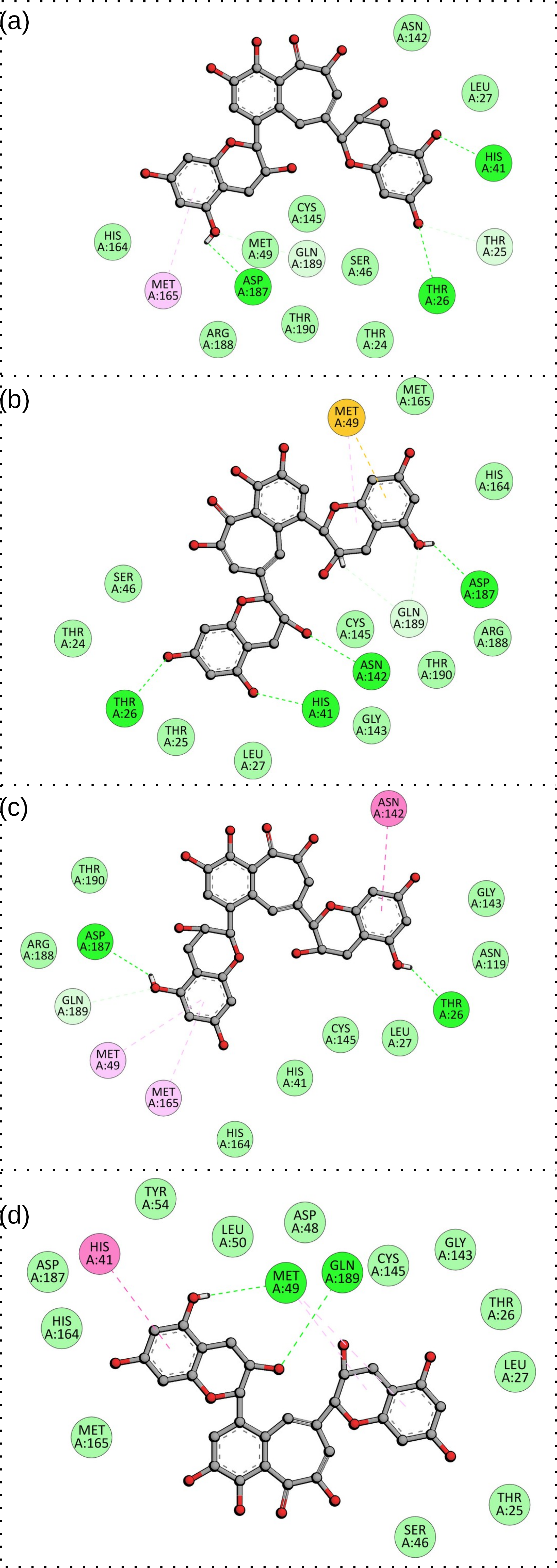
**

**Supplementary Fig. S3. Representation of the molecular interactions at the binding site of theaflavin-M^pro^ complex.** The binding poses are shown for time intervals (a) 20 ns, (b) 40 ns, (c) 60 ns, and (d) 80 ns.


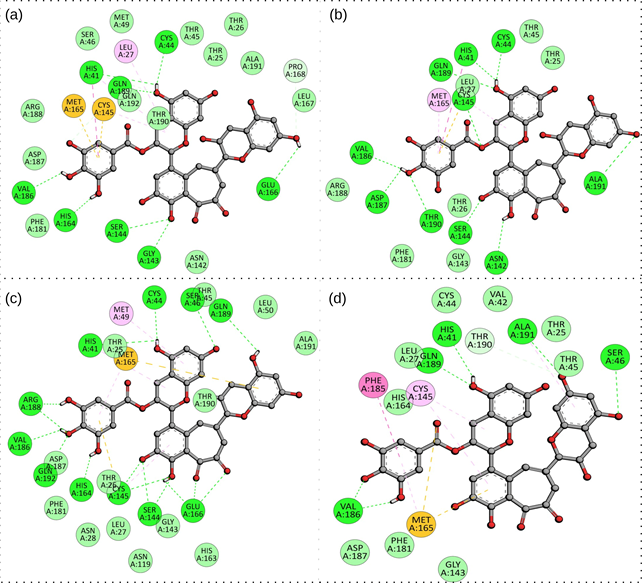


**Supplementary Fig. S4. Representation of the molecular interactions at the binding site of theaflavin 3-gallate-M^pro^ complex.** The binding poses are shown for time intervals (a) 20 ns, (b) 40 ns, (c) 60 ns, and (d) 80 ns.

**
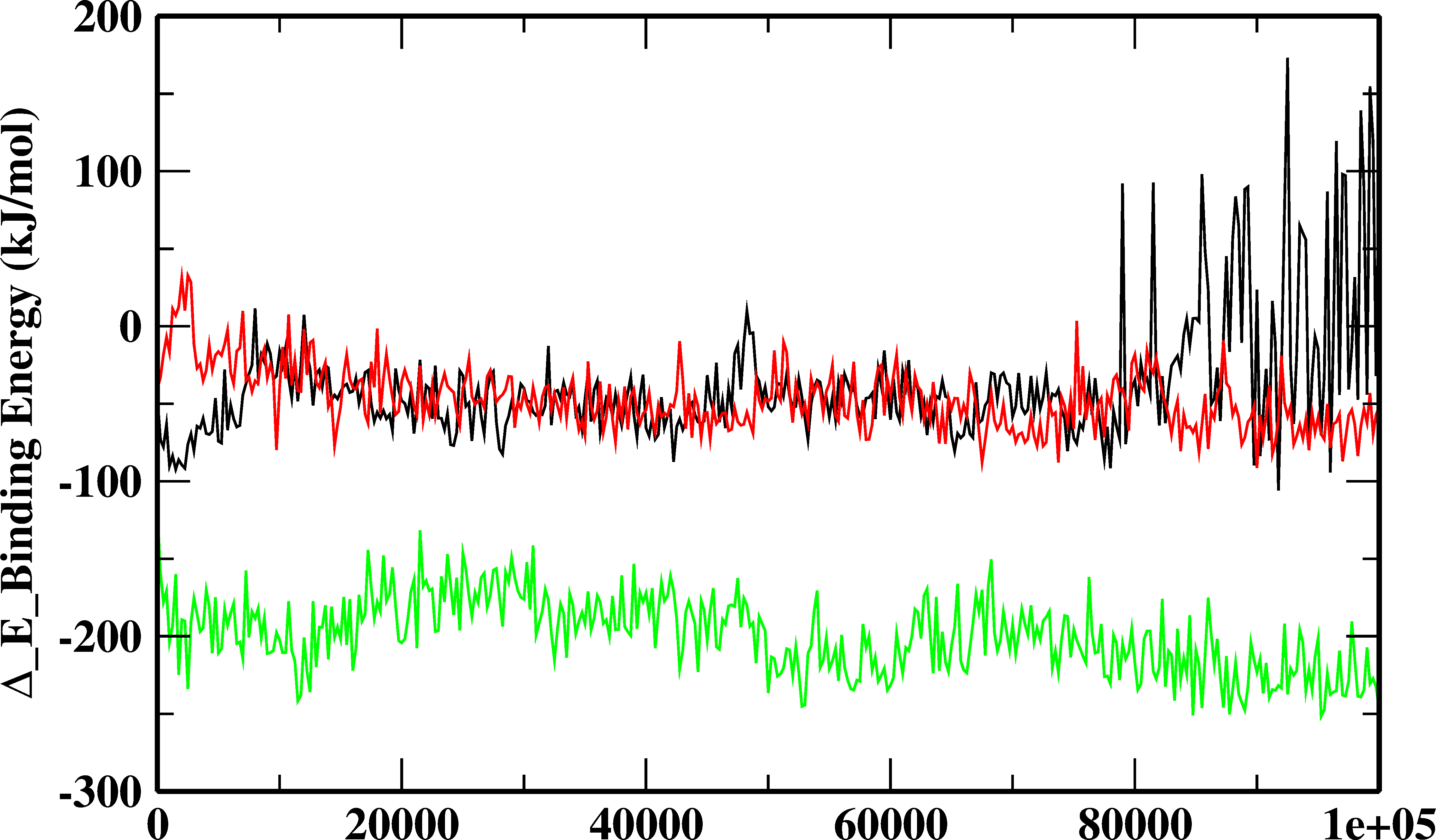
**

**Supplementary Fig. S5. The binding free energy calculated per frame by the MMPBSA method.** The color-coding scheme is as follows: GC373 (black), theaflavin (red), and theaflavin 3-gallate (green).

**
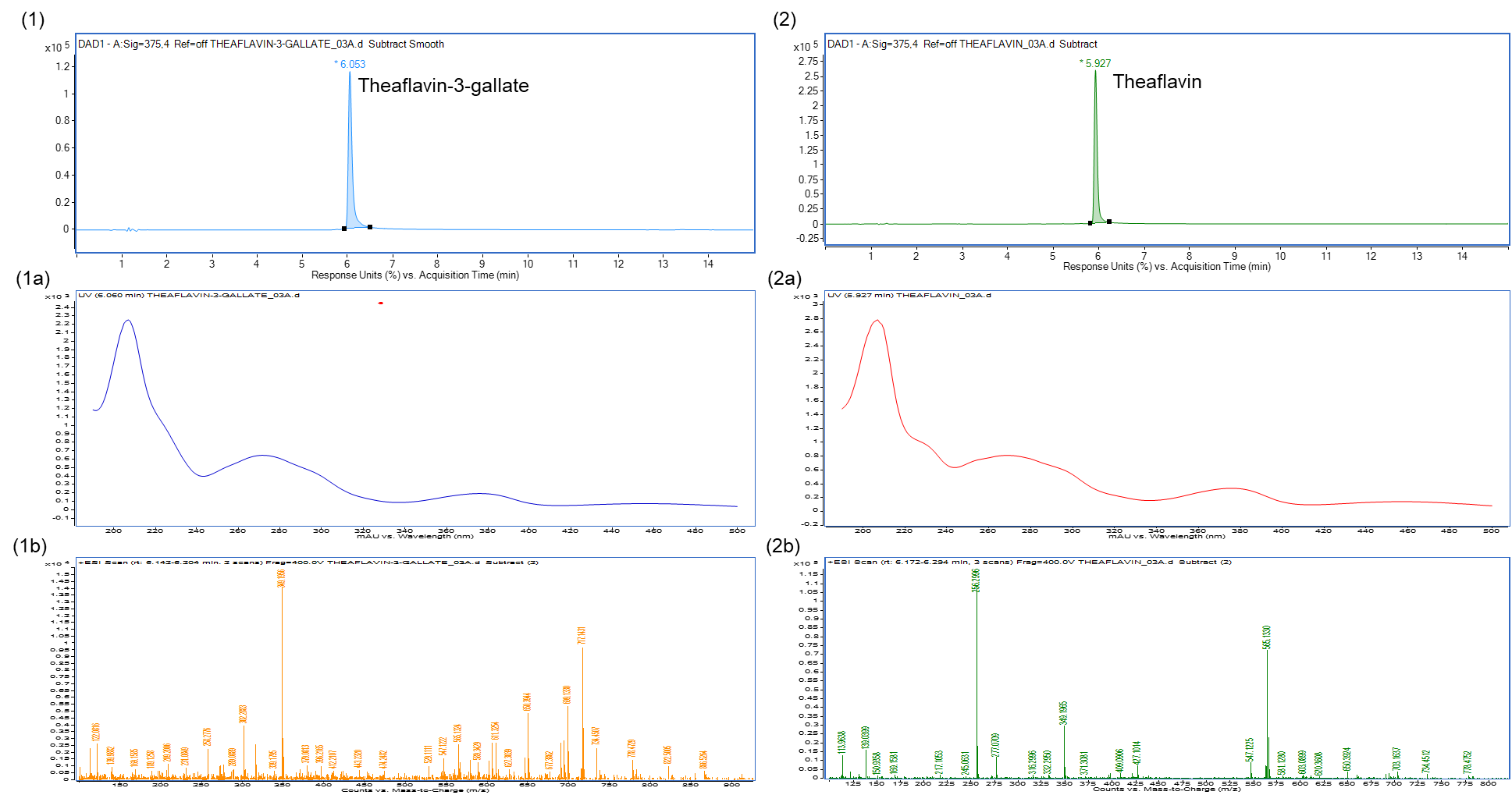
**

**Supplementary Fig. S6: LC-MS analysis of theaflavin 3-gallate and theaflavin.** (1) PDA chromatogram at 375 nm wavelength showing peaks at RT of 6.08 min and 5.92 min for theaflavin 3-gallate and theaflavin, respectively (1, 2). Mass spectrum (m/z) of the theaflavin-3-gallate and theaflavin (1a, 2a). UV spectrum of peaks for theaflavin 3-gallate and theaflavin peaks (1b, 2b).
